# Burden and Predictors of Malnutrition Among Indian Adolescents (10–19 Years): Insights From Comprehensive National Nutrition Survey Data

**DOI:** 10.3389/fpubh.2022.877073

**Published:** 2022-06-15

**Authors:** Raghavendra Pandurangi, Mahesh Kumar Mummadi, Sairam Challa, N. Samarasimha Reddy, Venkatesh Kaliaperumal, Chinta Khadar Babu, Usha Rani Telikicherla, Raghu Pullakandham, J. J. Babu Geddam, Rajkumar Hemalatha

**Affiliations:** ^1^Division of Public Health Nutrition, Indian Council of Medical Research (ICMR)-National Institute of Nutrition, Hyderabad, India; ^2^Division of Clinical Epidemiology, Indian Council of Medical Research (ICMR)-National Institute of Nutrition, Hyderabad, India; ^3^Division of Pathology and Microbiology, Indian Council of Medical Research (ICMR)-National Institute of Nutrition, Hyderabad, India; ^4^Division of Drug Toxicology, Indian Council of Medical Research (ICMR)-National Institute of Nutrition, Hyderabad, India; ^5^Indian Council of Medical Research (ICMR)-National Institute of Nutrition, Hyderabad, India

**Keywords:** adolescents, Indian, CNNS, malnutrition, stunting, overweight, thinness, predictors

## Abstract

**Introduction:**

Malnutrition among adolescents is a persistent problem with a profound impact on different dimensions of health. The objective of this analysis is to assess the burden of malnutrition (Stunting, Thinness, Overweight, and Obesity) and their associated socio-demographic factors among Indian adolescents (10–19 years) from the Comprehensive National Nutritional Survey (CNNS 2016-18) data.

**Methods:**

We used Individual-level data of 35,831 adolescents from the CNNS conducted in 2016–18 for this analysis. CNNS collected data on the nutritional status of adolescents along with socio-demographic variables from all states of India. Burden of stunting (Height for age Z score, HAZ < −2 SD), thinness (BMI for age Z score, BAZ < −2 SD), overweight (BAZ > 1 SD) and obesity (BAZ > 2 SD) were estimated for the entire country and individual states. A multivariable logistic regression analysis was used to assess the socio-demographic factors associated with stunting, thinness, and overweight.

**Results:**

CNNS collected data from 35,831 adolescents, of which 31,941 with BAZ scores, and 32,045 with HAZ scores were included in the final analysis. The burden of stunting and thinness among Indian adolescents was 27.4% (95% CI 26.4, 28.4%) and 24.4% (23.5, 25.4%), respectively. The burden of overweight and obesity was 4.8% (4.5, 5.1%) and 1.1% (0.9, 1.3%), respectively. Adolescents in the age group of 15–19 years (AOR 1.23, 95% CI 1.11, 1.36) compared to 10–14 years, females (AOR 1.20; 1.08, 1.33) compared to males, were at increased odds of getting stunted. Adolescents from lowest wealth index families (AOR 1.66; 1.33, 2.07) were at increased odds of thinness compared to peers of higher wealth index families. Adolescents of 10–14 years (AOR 1.26, 95% CI 1.06, 1.49) compared to 15–19 years, urban residents (AOR 1.43, 95% CI 1.19, 1.71) compared to rural residents, were at increased odds of overweight.

**Conclusion:**

Indian adolescents face the double burden of malnutrition that is undernutrition (stunting and thinness) alongside overnutrition (overweight and obesity) that are linked with socio-demographic factors. The National Nutritional Programs (POSHAN Abhiyan) should prioritize high-risk groups specifically older age group (15–19 years), females, and low wealth Index quintile families identified in this analysis.

## Introduction

Adolescence (aged 10–19 years) is an essential phase in a child's growth with rapid physical, psychological, and cognitive development (1, 2). The 1.2 billion global adolescent population is the largest cohort, making up 16% worldwide. In India, 253 million adolescents reside, translating to one-fifth of the global adolescent population (3). The health and wellness of the adolescents is important as 45% of potential skeletal growth, 15–25% of adult height gain, and 37% of total bone mass accumulation occur during this age (4, 5). Malnutrition during adolescence profoundly impacts the developmental processes (6). The short term complications of undernutrition (thinness or stunting) are being underweight, poor performance at school and risk of frequent infections. In the long term, undernutrition among adolescents is associated with poor general health, and less economic productivity (4, 5). On the other hand, over-nutrition contributes to the early development of non-communicable diseases such as diabetes, hypertension, coronary heart diseases, sleep apnoea, cancer (7).

In India, the burden of malnutrition is periodically estimated by nationally representative surveys like the National Family Health Survey (NFHS), the National Nutrition Monitoring Bureau (NNMB), the District Level Health Surveys (DLHS), the Annual Health Surveys (AHS), and the Rapid Survey of Children (RSoC). National Family Health Survey-4 reported the nutritional status of the 15–19 years age group using body mass index (BMI) as an indicator but not BMI-for-age z-scores (BAZ) (8). As per the NFHS-4 report, the burden of thinness (BMI <18.5 kg/m^2^) among adolescent boys and girls of 15–19 years age was 45 and 42%, respectively (8). The Rapid Survey of Children (RSoC) conducted in 2013–2014 estimated the burden of thinness (BMI <18.5 kg/m^2^) among adolescent girls of age 10–14 as 77% and that of 15–18 years age group as 45% (9).

In India, there are no nationally representative data on nutritional status of adolescents (10–19 years of age) that use comprehensive and appropriate anthropometric indicators. For adolescents, using the standard BMI-for age (BAZ) scores by World Health Organization (WHO) (10) or using BMI cut off scores for adolescents by Cole et al. would be more appropriate to describe the burden of malnutrition (11). The Comprehensive National Nutritional Survey (CNNS) conducted during 2016–18 collected the nutritional and sociodemographic variables among the children of 0–19 years (12). It is the only comprehensive data on the nutritional status of the adolescent age group in India. In the present context, with dearth of data on the nutritional status of adolescents by using appropriate anthropometric indicators, this secondary data analysis was planned using the CNNS data. In this secondary data analysis, we report the prevalence of undernutrition (Stunting, Thinness) and overnutrition (Overweight and Obesity) among 10–19-year-old adolescents in India, stratified by state, age, gender, and other socio-demographic variables. In addition, we assessed socio-demographic factors associated with stunting, thinness, and overweight.

## Methods

### Study Population and Sampling Design

The CNNS was conducted from February 2016 to October 2018 by the Ministry of Health and Family Welfare, Government of India, collaborating with United Nations Children's Fund (UNICEF) and the Population Council. The methodological details of the survey are available in detail in the CNNS report (12). Briefly, the CNNS collected data from three target age groups- 0–4 years, 5–9 years, and 10–19 years in all states of India. A multistage, stratified, probability proportional to size (PPS) survey design covering rural and urban households was used. Villages in the rural area and municipal wards in the urban area formed the primary sampling units (PSU). Census Enumeration Blocks (CEB) were the secondary sampling units in urban areas. The PSUs were selected by PPS, and households were selected randomly. Only one child or adolescent was chosen from each age group in each household. Adolescents with major physical deformity, cognitive disabilities, chronic illness (e.g., tuberculosis, cancer, liver, and renal disease), acute febrile or infectious illness, acute injury, ongoing fever, and pregnant adolescents were excluded. The present secondary data analysis needed no institutional ethical approval. However, Ethical approvals for the CNNS were obtained from the Population Council's Institutional Review Board, New York, and the Institutional Ethical Committee of Post Graduate Institute of Medical Education and Research, Chandigarh, India (PGI/IEC/2015/1508).

### Data Collection

The CNNS used a household and an individual questionnaire for all participants using the Computer-Assisted Personal Interviewing (CAPI) method in the principal languages of the state and/or English after obtaining informed consent from the caregiver and assent from the adolescent (12). For everyone, data were collected on age, gender, location/region, religion, social class, current educational status, mother's education, wealth index, and region of the country as per the standard definitions (Supplementary Table 1). Height and weight were collected using a Stadiometer and digital weighing scale, respectively. During the CNNS survey, anthropometry measurements were internally monitored, and a three tier system of data quality assurance mechanisms were implemented.

### Statistical Analysis

Individual-level data for 10–19 years collected from the CNNS was accessed for this secondary analysis. The age group 10–19 years included children who had completed 120 months till 228 months. The age group was classified as early adolescents (120–179 months) and late adolescents (180–228 months). World Health Organization (WHO) Anthro Plus software was used to derive the BMI-for-age z-score (BAZ) and Height-for-age z-score (HAZ) (13). WHO standard classifications were followed to define the outcome variables. Stunting was defined as HAZ < −2 standard deviation (SD), Thinness as BAZ < −2 SD, Overweight as BAZ > +1 SD, and Obesity as BAZ > +2SD (13). The proportion of adolescent children with stunting, thinness, overweight, and obesity, along with 95% confidence intervals (95% CI) was estimated at the national level and for each state and union territory. The estimated burden of stunting, thinness, overweight, and obesity for individual states and union territories are plotted on an Indian map using R studio.

**Figure 1:**
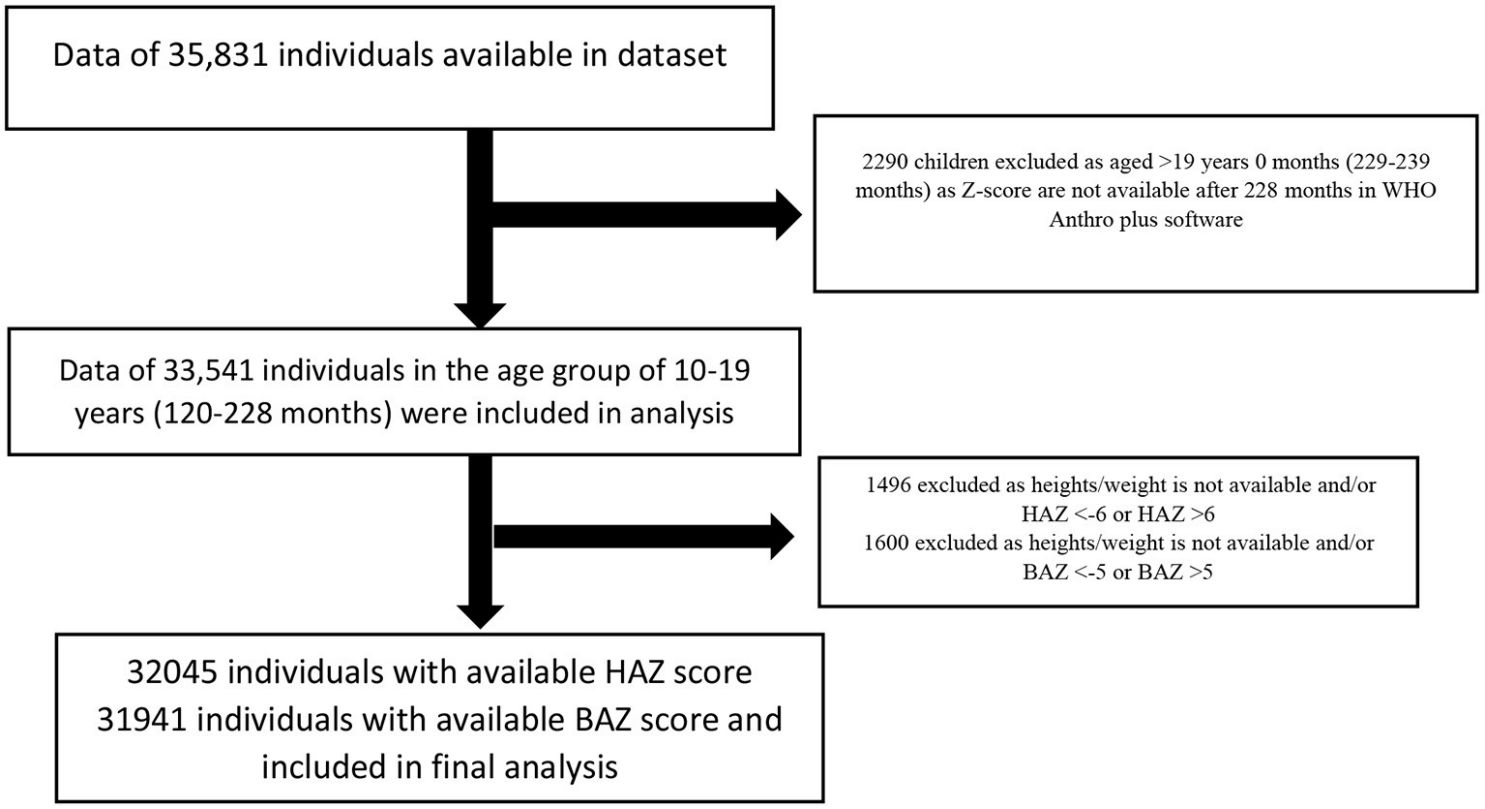
Flow diagram of children aged 10–19 years included in the analysis.

Data was collected from 35,831 adolescents during the CNNS survey, of which 31,941 adolescents had BAZ scores, and 32,045 had HAZ score and were included in the final analysis (Figure 1). The collected data included children aged 10 years and above (120 months onwards) till 19 years (239 months). As the z-scores were available in WHO references till the age of 19 years (228 months) only, the data of children aged 229–239 months was excluded for analysis. STATA version 13 (StataCorp LLC, College Station, TX, USA) was used for all statistical analyses. The association between socio-demographic characteristics and individual anthropometric indicators (Stunting, Thinness, and overweight) was assessed through a Univariate logistic regression analysis. The socio-demographic variables include age, gender, religion, residence, social class, maternal education, wealth index, and region of India. A multivariable logistic regression model examined the association between socio-demographic characteristics and individual anthropometric indicators. Variables with a *p*-value < 0.25 on univariate analysis, variables of contextual and clinical importance were included in the final model (14). A *p*-value < 0.05 was considered statistically significant.

## Results

### The Burden of Malnutrition in India

Stunting (HAZ < −2 SD) was seen in 8,877 Indian adolescents with a burden of 27.4% (95% CI 26.4, 28.4%). The burden of thinness was 24.4% (95% CI 23.5, 25.4%) as seen in 7,896 adolescents. The double burden of stunting and thinness in the same individual was seen in 8.6% (95% CI 8.3, 8.9%) adolescents, while the prevalence of co-existing stunting and overweight was seen in 0.8% (0.7, 0.9%). The burden of overweight and obesity among Indian adolescents was 4.8% (95% CI 4.5, 5.1%) and 1.1% (95% CI 0.9, 1.3%), respectively (Table 1).

**Table 1:** Burden of stunting (Height-for-age Z score < −2 SD), Thinness (BMI-for-age Z Score (BAZ) < −2 SD), Overweight (BAZ >1 SD), Obesity (BAZ >2 SD) among Indian children aged 10–19 years from Comprehensive national nutrition survey data collected during 2016–2018.

	**Children included in**	**Weighted**	**Children with altered**	**Weighted**
**Anthropometric measurement**	**analysis unweighted *N***	***N***	**anthropometry weighted (*n*)**	**proportion (95% CI)**
Stunting (HAZ < −2 SD)	32,045	32,412	8,877	27.4 (26.4, 28.4)
Thinness (BAZ < −2 SD)	31,941	32,296	7,896	24.4 (23.5, 25.4)
Overweight (BAZ >1 SD)	31,941	32,296	1,548	4.8 (4.5, 5.1)
Obesity (BAZ >2 SD)	31,941	32,296	347	1.1 (0.9, 1.3)
Stunting and thinness	31,941	32,296	2,776	8.6 (8.3, 8.9)
(HAZ < −2 SD and BAZ < −2 SD)				
Stunting and overweight	31,941	32,296	252	0.8 (0.7, 0.9)
(HAZ < −2 SD and BAZ > 1 SD)				

### Geographic Distribution of Malnutrition in India

**Figure 2:**
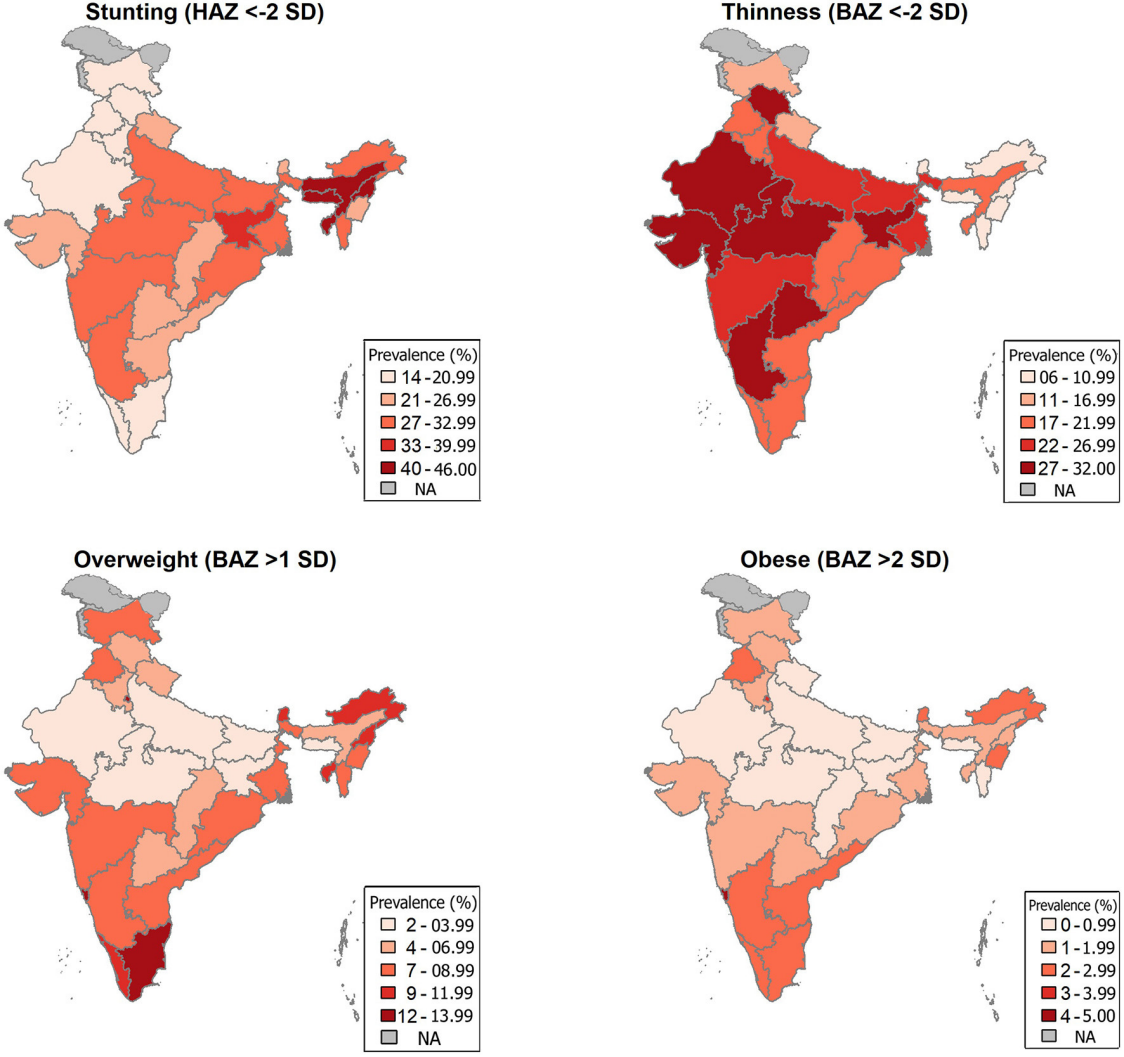
State-wise burden of Stunting, Thinness, Overweight, and Obesity among Indian children aged 10–19 years.

A higher prevalence of stunting was seen in north-eastern states—Assam (41.3, 95% CI 37.5, 45.2), Meghalaya (45.9, 95% CI 40.4, 51.6), Nagaland (41.8, 95% CI 36.8, 47.0) and Jharkhand (34.1, 95% CI 29.6, 38.9) compared to the national average (27.4 95% CI 26.4, 28.4). Seven states have thinness more than the national average (24.4, 95% CI 23.5, 25.4), two of which are from the Southern region—Telangana (29.1, 95% CI 25.7, 32.7), and Karnataka (27.3, 95% CI 23.6, 31.4). Overweight (India 4.8, 95% CI 4.5, 5.1) was seen to be more prevalent in Tamil Nadu (12.4, 95% CI 10.1, 15.1), Delhi (12.0, 95% CI 9.6, 14.9%), Goa (14.3, 95% CI 12.0, 16.9), Kerala (9.5, 95% CI 7.3, 12.3), Sikkim (9.3, 95% CI 6.9, 12.4), Arunachal Pradesh (11.1, 95% CI 8.9, 13.8), and Tripura (9.3, 95% CI 7.5, 11.5). States with a prevalence of overweight lower than the national average include Madhya Pradesh (1.7, 95% CI 1.1, 2.5), Uttar Pradesh (2.1, 95% CI 1.5, 3.0), Bihar (1.8, 95% CI 1.1, 3.0), and Jharkhand (1.9, 95% CI 1.2, 3.1). Obesity was found to be higher than the national average (1.1%, 95% CI 0.9, 1.3) in states like Goa (5.0%, 95% CI 3.7, 6.5), Delhi (3.3%, 95% CI 2.1, 5.0), Tamilnadu (2.6%, 95% CI 1.7, 3.8), Punjab (2.6%, 95% CI 1.8, 4.0), and Manipur (2.6%, 95% CI 1.7, 3.9) (Figure 2).

### Predictors of Stunting Among Adolescents in India Through Multivariate Regression Analysis

Adolescents in the age group of 15–19 years (AOR 1.23, 95% CI 1.11, 1.36), female gender (AOR 1.20, 95% CI 1.08, 1.33), Muslim religion (AOR 1.20, 95% CI 1.02, 1.42), scheduled caste (AOR 1.36, 95% CI 1.16, 1.59) and scheduled tribes (AOR 1.57, 95% CI 1.25, 1.98) were at increased odds of stunted growth compared to their counterparts (Table 2). Similarly, adolescents never attending the school (AOR 1.33, 95% CI 1.09, 1.63), belonging to the lowest wealth index (AOR 2.54, 95% CI 2.05, 3.14) were at increased odds of being stunted. Adolescents from all other parts of India were at increased odds of stunting compared to adolescents from the northern region of the Country (Table 2).

**Table 2:** Predictors of stunting among adolescent children (10–19 years) of India through Logistic regression model (weighted *N* = 32,412).

**Variable**	**Not stunted *n* (%)**	**Stunted *n* (%)**	**Unadjusted Odds ratio (95% CI)**	**Adjusted Odds ratio (95% CI)**	*P*-value*
**Age**					
10–14 years	13,397 (56.9)	4,619 (52)	Reference	Reference	-
15–19 years	10,139 (43.1)	4,258 (48)	1.22 (1.10–1.34)	1.23 (1.11–1.36)	0.000
**Gender**					
Male	11,993 (50.9)	4,119 (46.4)	Reference	Reference	-
Female	11,542 (49.1)	4,758 (53.6)	1.20 (1.09–1.32)	1.20(1.08–1.33)	0.001
**Residence**					
Urban	6,066 (25.7)	1,733 (19.5)	Reference	Reference	-
Rural	17, 469 (74.3)	7,144 (80.5)	1.43 (1.28–1.57)	0.93 (0.83–1.06)	0.261
**Religion**					
Hindu	19,074 (81)	7,094 (79.9)	Reference	Reference	-
Muslim	3,367 (14.3)	1,442 (16.3)	1.15 (0.97–1.36)	1.20 (1.02–1.42)	0.033
Christian	540 (2.3)	178 (2)	0.89 (0.69–1.14)	0.82 (0.59–1.15)	0.261
Others	554 (2.4)	163 (1.8)	0.79 (0.59–1.07)	1.11 (0.84–1.46)	0.473
**Social class**					
Others	4,842 (21.7)	1,227 (14.9)	Reference	Reference	-
Scheduled caste	5,227 (23.5)	2,101 (25.5)	1.59 (1.37–1.84)	1.36 (1.16–1.59)	0.000
Scheduled tribes	2,292 (10.3)	1,291 (15.6)	2.22 (1.76–2.81)	1.57 (1.25–1.98)	0.000
Other backward classes	9,890 (44.5)	3,630 (44)	1.45 (1.25–1.68)	1.30 (1.12–1.51)	0.001
**Schooling status**					
Currently in school	22,423 (95.3)	8,162 (92)	Reference	Reference	-
Never attended school	1,112 (4.7)	714 (8)	1.77 (1.47–2.12)	1.33 (1.09–1.63)	0.005
**Mother education**					
No schooling	11,925 (50.7)	5,394 (60.8)	2.43 (1.82–3.25)	1.26 (0.91–1.76)	0.164
1–5 years	3,287 (13.9)	1,314 (14.8)	2.15 (1.59–2.92)	1.35 (1.97–1.87)	0.074
6–12 years	7,264 (30.9)	1,972 (22.2)	1.46 (1.09–1.96)	1.11 (0.89–1.51)	0.527
>12 years	1,059 (4.5)	197 (2.2)	Reference	Reference	-
**Wealth index**					
Highest	5,288 (22.5)	1,018 (11.4)	Reference	Reference	-
High	4,932 (21)	1,404 (15.8)	1.48 (1.28–1.70)	1.33 (1.13–1.57)	0.001
Middle	4,602 (19.5)	1,858 (21)	2.10 (1.83–2.40)	1.81 (1.52–2.15)	0.000
Lower	4,533 (19.3)	2,041 (23)	2.34 (2.01–2.72)	1.88 (1.54–2.29)	0.000
Lowest	4,180 (17.7)	2,556 (28.8)	3.18 (2.70–3.74)	2.54 (2.05–3.14)	0.000
**Region of the country**					
North	3,610 (15.3)	806 (9.1)	Reference	Reference	-
Central	7,796 (33.1)	3,163 (35.6)	1.82 (1.52–2.17)	1.35 (1.13–1.60)	0.001
East	5,017 (21.3)	2,296 (25.9)	2.05 (1.72–2.44)	1.49 (1.23–1.80)	0.000
Nort-East	591 (2.5)	393 (4.5)	2.98 (2.39–3.72)	2.66 (2.06–3.43)	0.000
West	2,689 (11.5)	1,112 (12.5)	1.85 (1.54–2.22)	1.95 (1.63–2.35)	0.000
South	3, 832 (16.3)	1,107 (12.4)	1.29 (1.09–1.54)	1.33 (1.11–1.59)	0.002

^*^P < 0.05 is considered statistically significant.

### Predictors of Thinness Among Adolescents in India Through Multivariate Regression Analysis

Adolescents from other backward classes (AOR 1.26, 95% CI 1.08, 1.47), lowest wealth index families (AOR 1.66, 95% CI 1.33, 2.07), western India (AOR 1.21, 95% CI 1.02, 1.44) were at increased odds of thinness compared to their counterparts at a statistically significant level. The age group of 15–19 years (AOR 0.68, 95% CI 0.61, 0.76) compared to 10–14 years age group, Female gender (AOR 0.57, 95% CI 0.51, 0.63) compared to males were at decreased odds of thinness. Adolescents from eastern India (AOR 0.79, 95% CI 0.66, 0.93) and north-eastern India (AOR 0.59, 95% CI 0.43, 0.88) were at decreased odds of thinness compared to adolescents from northern India (Table 3; Supplementary Table 2).

**Table 3:** Predictors of Thinness among adolescent children (10–19 years) of India through Logistic regression model (weighted *N* = 32,296).

**Variable**	**Nothinness *n* (%)**	**Thinness *n* (%)**	**Unadjusted Odds ratio (95% CI)**	**Adjusted Odds ratio (95% CI)**	*P*-value*
**Age**					
10–14 years	12,989 (53.2)	4,948 (62.7)	Reference	Reference	-
15–19 years	11,411(46.8)	2,948 (37.3)	0.68 (0.61–0.75)	0.68 (0.61–0.76)	0.000
**Gender**					
Male	11,249 (46.1)	4,794 (60.7)	Reference	Reference	-
Female	13,151 (53.9)	3,102 (39.3)	0.55 (0.50–0.61)	0.57 (0.51–0.63)	0.000
**Residence**					
Urban	6,176 (25.3)	1,603 (20.3)	Reference	Reference	-
Rural	18,224 (74.7)	6,293 (79.7)	1.33 (1.18–1.49)	1.11 (0.98–1.26)	0.088
**Religion**					
Hindu	19,614 (80.4)	6,457 (81.8)	Reference	Reference	-
Muslim	3,601 (14.8)	1,193 (15.1)	1.01 (0.86–1.18)	1.04 (0.89–1.23)	0.599
Christian	593 (2.4)	120 (1.5)	0.62 (0.48–0.78)	0.81 (0.63–1.05)	0.111
Others	592 (2.4)	126 (1.6)	0.65 (0.51–0.81)	0.77 (0.60–0.98)	0.037
**Social class**					
Others	4,780 (20.8)	1,278 (17.3)	Reference	Reference	-
Scheduled caste	5,609 (24.4)	1,707 (23.1)	1.14 (0.98–1.32)	0.99 (0.84–1.17)	0.955
Scheduled tribes	2,758 (12.0)	801 (10.8)	1.09 (0.90–1.31)	0.89 (0.72–1.09)	0.269
Other backward classes	9,853 (42.8)	3,609 (48.8)	1.37 (1.17–1.60)	1.26 (1.08–1.47)	0.003
**Schooling status**					
Currently in school	23,029 (94.4)	7,448 (94.3)	Reference	Reference	-
Never attended school	1,371 (5.6)	448 (5.7)	1.01 (0.79–1.29)	0.94 (0.71–1.24)	0.654
**Mother education**					
>12 years	1,051 (4.3)	203 (2.6)	Reference	Reference	-
6–12 years	7,172 (29.4)	2,031 (25.7)	1.46 (1.15–1.87)	1.26 (0.98–1.63)	0.073
1–5 years	3,434 (14.1)	1,151 (14.6)	1.73 (1.34–2.24)	1.35 (1.01–1.80)	0.039
No schooling	12,744 (52.2)	4,511 (57.1)	1.83 (1.43–2.33)	1.40 (1.06–1.85)	0.016
**Wealth index**					
Highest	5,124 (21.0)	1,161 (14.7)	Reference	Reference	-
High	4,911 (20.1)	1,412 (17.9)	1.27 (1.11–1.45)	1.23 (1.06–1.43)	0.006
Middle	4,749 (19.5)	1,686 (21.3)	1.57 (1.34–1.83)	1.58 (1.31–1.89)	0.000
Lower	4,785 (19.6)	1,773 (22.5)	1.63 (1.40–1.91)	1.62 (1.31–2.00)	0.000
Lowest	4,831 (19.8)	1,864 (23.6)	1.70 (1.44–2.01)	1.66 (1.33–2.07)	0.000
**Region of the country**					
North	3,339 (13.7)	1,065 (13.5)	Reference	Reference	-
Central	8,218 (33.7)	2,715 (34.4)	1.04 (0.85–1.26)	0.85 (0.70–1.04)	0.119
East	5,498 (22.5)	1,791 (22.7)	1.02 (0.87–1.19)	0.79 (0.66–0.93)	0.005
North-East	814 (3.3)	171 (2.2)	0.66 (0.50–0.87)	0.59 (0.43–0.80)	0.001
West	2,758 (11.3)	1,014 (12.8)	1.15 (0.97–1.37)	1.21 (1.02–1.44)	0.028
South	3,773 (15.5)	1,140 (14.4)	0.95 (0.82–1.09)	0.92 (0.79–1.07)	0.298

^*^P < 0.05 is considered statistically significant.

### Predictors of Overweight Among Adolescents in India Through Multivariate Regression Analysis

Adolescents in the age group of 10–14 years (AOR 1.26, 95% CI 1.06, 1.49), residence in urban areas (AOR 1.43, 95% CI 1.19, 1.71), others (general) category of social class (AOR 1.30, 95% CI 1.07, 1.57), mothers' education more than 12 years (AOR 1.66, 95% CI 1.33, 2.06) highest wealth index families (AOR 2.11, 95% CI 1.59, 2.79) were at increased odds of overweight compared to their counterparts. Similarly, adolescents residing in north-eastern India (AOR 1.87, 95% CI 1.28, 2.74), eastern India (AOR 1.49, 95% CI 1.10, 2.01), and southern India (AOR 1.85, 95% CI 1.48, 2.30) were at increased odds of overweight compared to adolescents residing in north India (Table 4).

**Table 4:** Predictors of overweight among adolescent children (10–19 years) of India through Logistic regression model (weighted *N* = 32,296).

**Variable**	**No overweight *n* (%)**	**Overweight *n* (%)**	**Unadjusted Odds ratio (95% CI)**	**Adjusted Odds ratio (95% CI)**	*P*-value*
**Age**					
10–14 years	16,997 (55.3)	939 (60.7)	1.25 (1.06–1.46)	1.26 (1.06–1.49)	0.009
15–19 years	13,751 (44.7)	609 (39.3)	Reference	Reference	-
**Gender**					
Male	15,262 (49.6)	781 (50.4)	Reference	Reference	-
Female	15,486 (50.4)	767 (49.6)	0.97 (0.83–1.13)	1.01 (0.87–1.18)	0.883
**Residence**					
Urban	7,027 (22.8)	752 (48.6)	3.19 (2.65–3.84)	1.43 (1.19–1.71)	0.000
Rural	23,721(77.2)	796 (51.4)	Reference	Reference	-
**Religion**					
Hindu	24,891 (81)	1,180 (76.2)	Reference	Reference	-
Muslim	4,531 (14.7)	262 (16.9)	1.22 (0.95–1.56)	1.01 (0.80–1.27)	0.929
Christian	670 (2.2)	44 (2.9)	1.38 (0.91–2.11)	0.72 (0.47–1.11)	0.136
Others	656 (2.1)	62 (4)	1.98 (1.46–2.70)	1.31 (0.97–1.78)	0.080
**Social class**					
Others	5,583 (19.3)	475 (32.6)	1.75 (1.43–2.14)	1.30 (1.07–1.57)	0.008
Scheduled caste	7,032 (24.3)	284 (19.5)	0.83 (0.64–1.08)	1.04 (0.80–1.35)	0.770
Scheduled tribes	3,488 (12.0)	74 (5.1)	0.44 (0.30–0.64)	0.79 (0.54–1.15)	0.296
Other backward classes	12,837 (44.4)	624 (42.8)	Reference	Reference	-
**Schooling status**					
Currently in school	28,965 (94.2)	1,512 (97.7)	Reference	Reference	-
Never attended school	1,783 (5.8)	36 (2.3)	0.38 (0.19–0.75)	1.14 (0.57–2.27)	0.714
**Mother education**					
No schooling	16,866 (54.8)	388 (25.1)	0.26 (0.21–0.32)	0.63 (0.51–0.79)	0.000
1–5 years	4,374 (14.3)	211 (13.5)	0.55 (0.43–0.69)	0.79 (0.62–1.01)	0.064
6–12 years	8,460 (27.5)	743 (48.2)	Reference	Reference	-
>12 years	1,048 (3.4)	206 (13.2)	2.24 (1.78–2.82)	1.66 (1.33–2.06)	0.000
**Wealth index**					
Highest	5,556 (18.1)	729 (47.1)	3.60 (2.79–4.64)	2.11 (1.59–2.79)	0.000
High	5,924 (19.3)	399 (25.8)	1.85 (1.44–2.37)	1.41 (1.08–1.85)	0.011
Middle	6,208 (20.1)	226 (14.6)	Reference	Reference	-
Lower	6,419 (20.9)	139 (9)	0.59 (0.40–0.87)	0.74 (0.49–1.10)	0.133
Lowest	6,641 (21.6)	55 (3.5)	0.23 (0.14–0.37)	0.31 (0.18–0.53)	0.000
**Region of the country**					
North	4,267 (13.6)	237 (15.4)	Reference	Reference	-
Central	10,699 (34.8)	234 (15.0)	0.38 (0.27–0.54)	0.68 (0.50–0.92)	0.013
East	6,988 (22.7)	301 (19.4)	0.76 (0.55–1.03)	1.49 (1.10–2.01)	0.010
North-East	934 (3.0)	51 (3.3)	0.96 (0.68–1.36)	1.87 (1.28–2.74)	0.001
West	3,498 (11.4)	273 (17.7)	1.37 (1.04–1.81)	1.20 (0.93–1.55)	0.167
South	4,462 (14.5)	452 (29.2)	1.78 (1.42–2.22)	1.85 (1.48–2.30)	0.000

^*^P < 0.05 is considered statistically significant.

## Discussion

Our analysis has shown that among Indian adolescents, 27.4% were stunted, 24.4% were thin, 4.8 and 1.1% were overweight and obese, respectively. While stunting was higher in girls and the late adolescent age group (15–19 years), thinness was higher in boys and early adolescence (10–14 years). Overweight was marginally higher in boys than girls and early adolescence compared to late adolescence. The socio-demographic factors of adolescent malnutrition (stunting, thinness, and overweight) in India from our analysis include gender, wealth index, social class, religion, and maternal education.

### Stunting and Its Predictors

In the present study, the prevalence of stunting was higher in late adolescence (30% in late adolescence vs. 25.6% in early adolescence). According to the Global School-Based survey conducted in 57 Low-Middle-Income countries (LMICs) on children aged 12–15 years, 14.6% of adolescents in India were stunted (15). Our analysis has shown higher estimates of stunting, which could be due to the inclusion of 15–19-year-old subjects, where the prevalence of stunting was 4% higher than 10–14-year adolescents. Among Indian adolescents in the 15–19 years age group, Bhargava et al. reported that stunting was 29.1% (95% CI 28.6–29.6) in NFHS-3 (2005-06), which increased to 34.1% (95% CI 33.9–34.4) in NFHS-4 (2015-16) (16). Our estimates from CNNS data on stunting are lower than both NFHS-3 and NFHS-4. The lower estimates could be because NFHS represents only the late adolescence (15–19 years), while CNNS includes both early and late adolescence (10–19 years).

The burden of stunting among girls was higher than boys, corroborating with previous estimates by Bhargava et al. on NFHS data (16). Indian boys and girls had similar growth faltering compared to the WHO median until 14 years, and after this age, girls grew slower, leading to a higher burden of stunting. This was demonstrated from the review of National nutrition monitoring bureau (NNMB) data from their surveys in 1975-79 and 2012-13 (17). Stunting among Indian adolescents is lower compared to adolescents of other countries in Asia [48% in rural Bangladesh (18) and 23.7% in Malaysia (19)] and other African countries [12.2% in Ethiopian adolescent girls (20) and 9.7% in Somalian refugee camps (6)]. No significant difference between gender at any age was reported in contrast to the present findings (21). The risk of stunting increases with the increase of age. This is attributable to different chronic factors such as food security, education, gender disparities, women's health (22). Furthermore, repeated infections and environmental stress apart from poor nutrition collectively from intrauterine stages through adolescence culminate in stunting (22). The odds of being stunted were high among North-eastern India, lowest wealth index families, scheduled tribes, scheduled caste, never attending the school, and Muslim religion consistent with predictors from the secondary analysis on NFHS data (16). Increased odds of stunting in North-eastern India compared to northern India may be attributable to the lower growth potential of the hilly north-eastern region. At the same time, the other factors are all related to vulnerability, affordability, and deprivation.

### Thinness and Its Predictors

In the current study, thinness was higher in boys (29.9%) than girls (18.9%). In NFHS-3, the reported prevalence of thinness was 22.3% (boys) and 9.9% (girls), which subsequently decreased to 16.5 and 9% in NFHS-4 in boys and girls, respectively. In many neighboring countries, thinness among boys is higher than girls, viz Indonesia (11 vs. 5%), Bangladesh (19.6 vs. 15.4%), and Nepal (37.8 vs. 26.2%) (23–25). This pattern of higher prevalence of thinness among boys is similar to aggregates based on 57 LMICs (6.7% in boys and 4.5% in girls) (15). It might be attributed to the increased gain in height among boys compared to girls leading to higher levels of thinness.

In the present study, the prevalence of thinness was 27.6% in early adolescence and 20.9% in late adolescence, which was similar to findings by Kumar P et al. observed in adolescents of Bihar and Uttar Pradesh (26). This could be due to increased growth spurt in the early adolescent stage compared to the late adolescent stage (20). This might also be due to higher calorie intake deficiencies among early adolescents than late adolescents (27). In the current study, apart from being a boy or belonging to early adolescence, the odds of being thin were high among those from—other backward classes, lowest wealth index families, rural residence, north-eastern India, or whose mothers had no schooling, all of which were associated with increased risk of undernutrition.

### Overweight and Its Predictors

Our analysis has shown a marginally higher prevalence of overweight (BAZ > 1SD) among boys (4.9%) compared to girls (4.7%), with no statistically significant difference. The prevalence rates of overweight reported in NFHS-4 were slightly higher than CNNS data, and there was a marginal increase between NFHS-3 (3.0% in boys and 4.3% in girls) and NFHS-4 (6.2% in boys and 4.9% in girls) (16). The burden of overweight is lower in India compared to international estimates. Systematic analysis of 1,769 studies has shown that in developing countries, the prevalence of overweight among children and adolescents has increased from 8.1 to 12.9% in boys and 8.4 to 13.4% in girls between 1980 and 2013 (28). Late adolescents (4.1%) had a lower burden of overweight than early adolescents (5.2%) and had statistically significant lower odds of being overweight. Adolescents residing in urban areas and those of the highest wealth index families were at increased odds of being overweight compared to their counterparts. The odds of being overweight were higher among adolescents from the south and west India compared to those residing in Northern India.

The literature about coexisting stunting with thinness or obesity is sparsely available amongst adolescents in India. The UDAYA study conducted in Uttar Pradesh and Bihar states reports that the co-existence of stunting with thinness was seen in 11 percent of boys and 8 percent of early adolescent girls (26).

The strength of the present analysis is the use of CNNS data, a large, quality-controlled, recent, and representative national sample. We have used BAZ scores, an appropriate indicator for reporting nutritional status among adolescents unlike BMI which is appropriate for adults and used in NFHS surveys. We have not analyzed and triangulated other forms of malnutrition, including the micronutrient deficiencies and co-existence of different forms of malnutrition which is a limitation of this analysis. Another limitation of this analysis is that the socio-demographic predictors of malnutrition among adolescents needs to be triangulated with clinical characteristics and dietary intakes. Since adolescent malnutrition especially stunting and thinness, are high, correcting these in this age group could be the game-changer in breaking the intergenerational cycle of malnutrition. With the identified burden of overweight and obesity, interventions focusing on the early lifestyle changes in this age group must be taken up through already existing national health programs. Identification of state-specific determinants of malnutrition coupled with effective interventional strategies can benefit in a targeted reduction in the burden of malnutrition. Growth monitoring and nutritional surveillance like quarterly anthropometric assessment can contribute to the real-time correction of malnutrition among adolescents. School health programs and POSHAN Abhiyan (Prime Minister's Overarching Scheme for Holistic Nourishment) must be linked with the periodic anthropometric assessment of this age group.

## Conclusion

Among Indian adolescents, there is the burden of thinness, stunting, and overweight, indicating the double burden of malnutrition. Socio-demographic factors of malnutrition identified in this analysis include gender (female), religion (Muslim, Christian), social class (Scheduled castes and scheduled tribes), not attending school, no/less maternal education, poor wealth index families. These factors can prioritize interventional strategies at the district and state levels to eliminate malnutrition. Adolescence presents a final opportunity for correction of malnutrition before the child enters adulthood. Nutritional surveillance in this age group coupled with targeted nutritional programs for undernutrition and lifestyle changes for overweight and obesity will correct malnutrition in this crucial age group.

## Data Availability Statement

The original contributions presented in the study are included in the article/Supplementary Material, further inquiries can be directed to the corresponding author.

## Ethics Statement

Ethical approvals for the CNNS were obtained from the Population Council's Institutional Review Board, New York, and the Institutional Ethical Committee of Post Graduate Institute of Medical Education and Research, Chandigarh, India (PGI/IEC/2015/1508).

## Author Contributions

RPa, MM, and SC conceptualized the article, interpreted the results, and contributed to manuscript preparation. UT and VK was involved in manuscript writing. NR was involved in conceptualizing the article, performing statistical analysis, interpreting the results, and writing the manuscript. CK performed the statistical analysis. JG, RPu, and RH critically reviewed the manuscript and had the final decision in processing the manuscript. All authors contributed to the article and approved the submitted version.

## Conflict of Interest

The authors declare that the research was conducted in the absence of any commercial or financial relationships that could be construed as a potential conflict of interest.

## Publisher's Note

All claims expressed in this article are solely those of the authors and do not necessarily represent those of their affiliated organizations, or those of the publisher, the editors and the reviewers. Any product that may be evaluated in this article, or claim that may be made by its manufacturer, is not guaranteed or endorsed by the publisher.
