# Supplementary material for: Burden and Predictors of Malnutrition Among Indian Adolescents (10–19 Years): Insights From Comprehensive National Nutrition Survey Data
Source: Front Public Health. 2022 Jun 15;10:877073. doi: 10.3389/fpubh.2022.877073 (PMC9240318; doi:10.3389/fpubh.2022.877073)
Supplement: Supplementary file 1 [file Table_1.pdf]

Supplementary files

Supplementary table 1: standard definitions for socio-demographic variables used in the analysis

Variable	Categories
**Age (in years)**	10-14 years (120-179 months)
	15-19 years (180-228 months)
**Gender**	Male
	Female
**Residence**	Urban
	Rural
**Religion**	Hindu
	Muslim
	Christian
	Others (Sikh, Jain, etc..)
**Social Class**	Others (general)
	Other backward Classes (Socially and educationally backward classes of citizens recognised by the government as per Indian constitution)
	Scheduled Caste (Scheduled castes are communities in India which have been recognised by the government as per Indian constitution)
	Scheduled Tribes (Scheduled tribes are the tribal communities recognised by the government as per Indian constitution)
**Schooling Status**	Currently In school
	Never attended school
**Mother education**	No schooling
	1-5 years
	6-12 years
	>12 years
**Wealth Index (quintiles based on the possession of a number of common household items)**	Highest
	High
	Middle
	Lower
	Lowest
**Region of the Country**	**North** (Delhi, Haryana, Himachal Pradesh, Jammu & Kashmir, Punjab, Rajasthan, Uttarakhand)
	**Central** (Madhya Pradesh, Chhattisgarh, Uttar Pradesh)
	**East** (Bihar, Jharkhand, West Bengal, Odisha)
	**Nort-East** (Arunachal Pradesh, Assam, Manipal, Mizoram, Meghalaya, Nagaland, Sikkim, Tripura)
	**West** (Goa, Gujarat, Maharashtra)
	**South** (Andhra Pradesh, Karnataka, Kerala, Tamil Nadu, Telangana)

Supplementary table 2: Burden of Stunting, Thinness and Overweight among different demographic and socio-economic characteristics of Indian adolescents aged 10-19 years from CNNS survey during 2016-2018

**Variable**	Stunted<br>% (95% CI)	Thinness<br>% (95% CI)	Overweight<br>% (95% CI)
**Age**			
10-14 years	25.6 (24.4, 27)	27.6 (26.3, 28.9)	5.2 (4.8, 5.7)
15-19 years	30 (28.1, 31.1)	20.5 (19.3, 21.9)	4.2 (3.8, 4.8)
**Gender**			
Male	25.6 (24.3, 27)	29.9 (28.5, 31.3)	4.9 (4.4, 5.3)
Female	29.2 (27.8, 30.6)	19.1 (18, 20.3)	4.7 (4.2, 5.3)
**Residence**			
Urban	22.2 (21, 23.5)	20.6 (19.4, 21.8)	9.7 (8.9, 10.5)
Rural	29 (27.8, 30.3)	25.7 (24.5, 26.8)	3.2 (2.9, 3.6)
**Religion**			
Hindu	27.1 (26, 28.2)	24.8 (23.8, 25.8)	4.5 (4.2, 4.9)
Muslim	30 (27.5, 32.6)	24.9 (22.6, 27.4)	5.5 (4.5, 6.6)
Christian	24.8 (21, 29)	16.8 (13.6, 20.7)	6.2 (4.6, 8.2)
Others	22.7 (18.8, 27.2)	17.5 (14.3, 21.3)	8.6 (6.7, 11)
**Social Class**			
Others	20.2 (18.5, 22)	21.1 (19.4, 23)	7.8 (6.9, 8.8)
Scheduled Caste	28.7 (26.6, 30.8)	23.3 (21.5, 25.2)	3.9 (3.2, 4.7)
Scheduled Tribes	36 (32.9, 39.3)	22.5 (20, 25.2)	2.1 (1.6, 2.8)
Other backward Classes	26.9 (25.3, 28.4)	26.8 (25.3, 28.3)	4.6 (4.1, 5.2)
**Schooling Status**			
Currently In school	26.7 (25.7, 27.8)	24.4 (23.5, 25.4)	5 (4.6, 5.3)
Never attended school	39.1 (34.2, 44.3)	24.6 (20.4, 29.4)	2 (1, 3.8)
**Mother education**			
No schooling	31.1 (29.7, 32.7)	26.1 (24.8, 27.6)	2.2 (1.9, 2.6)
1-5 years	28.6 (26.1, 31.1)	25.1 (23, 27.4)	4.6 (3.8, 5.6)
6-12 years	21.4 (20, 22.8)	22.1 (20.7, 23.5)	8.1 (7.3, 9)
>12 years	15.7 (12.8, 19)	16.2 (13.4, 19.4)	16.4 (14, 19.3)
**Wealth Index**			
Highest	16.1 (14.9, 17.5)	18.5 (17.2, 19.9)	11.6 (10.6, 12.7)
High	22.2 (20.5, 23.9)	22.3 (20.7, 24)	6.3 (5.5, 7.3)
Middle	28.8 (26.7, 31)	26.2 (24.1, 28.4)	3.5 (2.8, 4.4)
Lower	31 (28.7, 33.5)	27 (24.9, 29.3)	2.1 (1.6, 2.8)
Lowest	38 (35.3, 40.7)	27.8 (25.4, 30.4)	0.8 (0.5, 1.2)
**Region of the Country**			
North	18.3 (16.8, 19.8)	24.2 (22.5, 26)	5.4 (4.7, 6.1)
Central	28.9 (26.8, 31)	24.8 (23, 26.8)	2.1 (1.7, 2.7)
East	31.4 (29.2, 33.6)	24.6 (22.6, 26.7)	4.1 (3.4, 4.9)
North-East	29.3 (27.1, 31.6)	26.9 (24.8, 29.1)	7.2 (6.1, 8.5)
West	22.4 (20.9, 24.1)	23.2 (21.7, 24.8)	9.2 (8.2, 10.3)

South	40 (37.1, 42.9)	17.4 (15.2, 19.7)	5.2 (4.2, 6.4)

Supplementary table-3: State-wise burden of stunting among Indian adolescents aged 10-19 years from CNNS survey during 2016-2018

State	Unweighted N	Weighted N	Stunted N (Weighted)	Weighted proportion	95% CI	
Meghalaya	880	63	29	45.9%	40.4%	51.6%
Nagaland	973	37	15	41.8%	36.8%	47.0%
Assam	1,272	717	296	41.3%	37.5%	45.2%
Tripura	937	56	22	40.2%	36.5%	44.0%
Jharkhand	954	1209	412	34.1%	29.6%	38.9%
Bihar	1,251	3262	1049	32.2%	28.7%	35.9%
Maharashtra	1,756	2402	752	31.3%	28.4%	34.4%
Arunachal Pradesh	987	19	6	30.1%	26.8%	33.7%
West Bengal	1,078	1943	582	30.0%	25.5%	34.8%
Madhya Pradesh	1,050	2994	895	29.9%	25.6%	34.5%
Mizoram	900	25	7	29.0%	25.8%	32.5%
Uttar Pradesh	1,690	7236	2078	28.7%	26.2%	31.4%
Odisha	1,126	900	253	28.1%	24.9%	31.4%
Karnataka	795	1141	319	27.9%	24.1%	32.0%
**India**	**32,045**	**32412**	**8877**	**27.40%**	**26.40%**	**28.40%**
Manipur	1,043	57	15	26.5%	23.3%	29.9%
Chhattisgarh	967	729	190	26.0%	22.9%	29.5%
Gujarat	919	1374	355	25.9%	22.7%	29.3%
Andhra Pradesh	1,014	1112	287	25.8%	22.5%	29.5%
Sikkim	909	9	2	24.0%	20.4%	28.0%
NCT of Delhi	1,368	385	82	21.3%	18.1%	24.9%
Uttarakhand	970	259	55	21.2%	18.2%	24.5%
Telangana	853	659	136	20.7%	17.8%	23.9%
Himachal Pradesh	1,055	312	64	20.4%	15.9%	25.8%
Tamil Nadu	1,677	1446	281	19.5%	16.6%	22.6%
Jammu and Kashmir	1,041	256	49	19.1%	16.2%	22.3%
Rajasthan	1,109	1964	354	18.0%	15.4%	20.9%
Goa	913	25	4	17.2%	14.6%	20.2%
Punjab	892	619	101	16.3%	13.3%	20.0%
Haryana	952	621	101	16.3%	13.7%	19.3%
Kerala	714	580	83	14.4%	11.6%	17.6%

Supplementary table-4: State-wise burden of thinness among Indian adolescents aged 10-19 years from CNNS survey during 2016-2018

State	Unweighted N	Weighted N	Thinness N (Weighted)	Weighted proportion	95% CI	
Madhya Pradesh	1,048	2984	965	32.3%	28.2%	36.8%
Himachal Pradesh	1,052	312	97	31.2%	25.8%	37.1%
Gujarat	912	1359	412	30.3%	27.1%	33.8%
Rajasthan	1,104	1955	572	29.2%	26.0%	32.7%
Telangana	847	655	190	29.1%	25.7%	32.7%
Jharkhand	954	1208	350	29.0%	24.6%	33.9%
Karnataka	793	1135	310	27.3%	23.6%	31.4%
West Bengal	1,077	1941	493	25.4%	21.4%	29.9%
Maharashtra	1,748	2387	596	25.0%	22.2%	27.9%
**India**	**31,941**	**32296**	**7896**	**24.40%**	**23.50%**	**25.40%**
Bihar	1,243	3246	780	24.0%	20.9%	27.4%
Uttar Pradesh	1,686	7221	1614	22.4%	20.1%	24.7%
Goa	910	25	5	21.8%	18.9%	25.0%
NCT of Delhi	1,369	386	81	21.1%	18.0%	24.6%
Tamil Nadu	1,674	1445	302	20.9%	18.0%	24.2%
Haryana	949	618	128	20.7%	17.8%	24.0%
Kerala	712	579	119	20.6%	17.4%	24.2%
Assam	1,268	720	146	20.3%	17.4%	23.5%
Andhra Pradesh	1,005	1100	219	19.9%	16.9%	23.2%
Odisha	1,120	895	168	18.8%	16.1%	21.8%
Chhattisgarh	966	728	135	18.6%	15.7%	21.8%
Punjab	893	620	113	18.2%	14.7%	22.2%
Tripura	931	55	9	16.8%	14.2%	19.7%
Uttarakhand	971	259	40	15.4%	12.8%	18.4%
Jammu and Kashmir	1,034	255	34	13.4%	11.0%	16.1%
Nagaland	976	37	4	10.6%	7.1%	15.7%
Sikkim	906	9	1	10.4%	7.5%	14.2%
Arunachal Pradesh	977	19	2	7.9%	6.2%	10.1%
Meghalaya	878	63	4	7.0%	4.8%	10.1%
Manipur	1,039	57	4	6.3%	4.7%	8.5%

Mizoram	899	25	2	6.3%	4.7%	8.4%

Supplementary table-5: State-wise burden of Overweight among Indian adolescents aged 10-19 years from CNNS survey during 2016-2018

State	Unweighted N	Weighted N	Overweight N (Weighted)	Weighted proportion	95% CI	
Goa	910	25	4	14.3%	12.0%	16.9%
Tamil Nadu	1,674	1445	179	12.4%	10.1%	15.1%
NCT of Delhi	1,369	386	46	12.0%	9.6%	14.9%
Arunachal Pradesh	977	19	2	11.1%	8.9%	13.8%
Nagaland	976	37	4	9.6%	7.3%	12.6%
Kerala	712	579	55	9.5%	7.3%	12.3%
Tripura	931	55	5	9.3%	7.5%	11.5%
Sikkim	906	9	1	9.3%	6.9%	12.4%
Punjab	893	620	56	9.1%	7.2%	11.4%
Andhra Pradesh	1,005	1100	97	8.9%	6.9%	11.3%
Manipur	1,039	57	5	8.7%	7.0%	10.7%
Jammu and Kashmir	1,034	255	22	8.6%	6.9%	10.6%
Mizoram	899	25	2	7.9%	6.2%	10.0%
Odisha	1,120	895	71	7.9%	6.3%	9.9%
Gujarat	912	1359	107	7.9%	6.1%	10.1%
West Bengal	1,077	1941	147	7.6%	5.7%	10.0%
Karnataka	793	1135	84	7.4%	5.7%	9.5%
Maharashtra	1,748	2387	163	6.8%	5.5%	8.5%
Telangana	847	655	37	5.7%	4.2%	7.5%
Himachal Pradesh	1,052	312	17	5.5%	3.2%	9.4%
Uttarakhand	971	259	13	5.2%	3.9%	6.9%
**India**	**31,941**	**32296**	**1548**	**4.8%**	**4.5%**	**5.1%**
Haryana	949	618	28	4.5%	3.4%	6.0%
Chhattisgarh	966	728	31	4.2%	2.8%	6.4%
Assam	1,268	720	30	4.2%	3.0%	5.9%
Meghalaya	878	63	2	3.5%	2.2%	5.5%
Rajasthan	1,104	1955	54	2.8%	1.9%	4.1%
Uttar Pradesh	1,686	7221	153	2.1%	1.5%	3.0%
Jharkhand	954	1208	24	1.9%	1.2%	3.1%
Bihar	1,243	3246	59	1.8%	1.1%	3.0%
Madhya Pradesh	1,048	2984	50	1.7%	1.1%	2.5%

Supplementary table-6: State-wise burden of Obesity among Indian adolescents aged 10-19 years from CNNS survey during 2016-2018

State	Unweighted N	Weighted N	Obesity N (Weighted)	Weighted proportion	95% CI	
Goa	910	25	1	5.0%	3.7%	6.5%
NCT of Delhi	1,369	386	13	3.3%	2.1%	5.0%
Punjab	893	620	16	2.6%	1.8%	4.0%
Manipur	1,039	57	1	2.6%	1.7%	3.9%
Tamil Nadu	1,674	1445	37	2.6%	1.7%	3.8%
Kerala	712	579	13	2.3%	1.4%	3.9%
Arunachal Pradesh	977	19	0	2.3%	1.3%	3.9%
Andhra Pradesh	1,005	1100	24	2.2%	1.1%	4.2%
Sikkim	906	9	0	2.1%	1.1%	3.9%
Karnataka	793	1135	24	2.1%	1.3%	3.3%
Tripura	931	55	1	2.0%	1.3%	3.1%
West Bengal	1,077	1941	37	1.9%	1.2%	3.0%
Odisha	1,120	895	17	1.9%	1.2%	2.8%
Maharashtra	1,748	2387	43	1.8%	1.1%	3.0%
Gujarat	912	1359	24	1.8%	1.0%	3.2%
Telangana	847	655	11	1.7%	1.0%	3.0%
Jammu and Kashmir	1,034	255	4	1.7%	1.0%	2.9%
Nagaland	976	37	1	1.6%	0.7%	3.8%
Assam	1,268	720	10	1.3%	0.6%	2.9%
Haryana	949	618	8	1.3%	0.7%	2.3%
Himachal Pradesh	1,052	312	4	1.1%	0.6%	2.3%
**India**	**31,941**	**32296**	**347**	**1.1%**	**0.9%**	**1.3%**
Uttarakhand	971	259	3	1.0%	0.6%	1.8%
Mizoram	899	25	0	1.0%	0.6%	1.8%
Chhattisgarh	966	728	6	0.8%	0.4%	1.6%
Uttar Pradesh	1,686	7221	29	0.4%	0.2%	1.0%
Rajasthan	1,104	1955	6	0.3%	0.1%	0.7%
Madhya Pradesh	1,048	2984	8	0.3%	0.1%	0.6%
Jharkhand	954	1208	3	0.2%	0.1%	0.9%
Meghalaya	878	63	0	0.2%	0.1%	0.5%
Bihar	1,243	3246	3	0.1%	0.0%	0.4%
